# Omega-3 polyunsaturated fatty acids and gut microbiota

**DOI:** 10.1097/MCO.0000000000001176

**Published:** 2025-10-29

**Authors:** Mark A. Hull, Hanyang Sun

**Affiliations:** Leeds Institute of Medical Research, University of Leeds, Leeds UK

**Keywords:** docosahexaenoic acid, eicosapentaenoic acid, metabolome, microbiome, short-chain fatty acids

## Abstract

**Purpose of review:**

Oral intake of *n* (omega)-3 polyunsaturated fatty acids (PUFAs) is associated with changes to gut microbiota. We review recent findings from 2024 onwards, which build the scientific case that changes to bacterial abundance, and their metabolites, contribute to the health benefits associated with *n*-3 PUFAs.

**Recent findings:**

There are now multiple studies in rodent disease models that demonstrate that *n*-3 PUFAs do not significantly alter bacterial diversity but, instead, alter abundance of several species that are implicated in short-chain fatty acid synthesis, in a model-specific manner. Limited intervention studies in humans, backed by larger observational studies, concur with the preclinical findings. Importantly, faecal transplantation experiments have confirmed that *n*-3 PUFA-induced changes to gut microbiota are causally related to reversal of the disease phenotype in two rodent models. In-vitro colonic models are now being used to understand the mechanism(s) underlying *n*-3 PUFA-induced changes to the gut microbiota and metabolome.

**Summary:**

Despite emerging proof that the gut microbiota contributes to *n*-3 PUFA activity in animal models, human data are sparse. It remains unclear how *n*-3 PUFAs affect changes to the gut microbiota or whether *n*-3 PUFA metabolism by gut microbes contributes to the host metabolome.

## INTRODUCTION

Treatment with high-dose *n* (omega)-3 polyunsaturated fatty acids (PUFAs) eicosapentaenoic acid (EPA) and docosahexaenoic acid (DHA) is licensed for cardiovascular risk reduction in high-risk individuals and in individuals with resistant hypertriglyceridaemia in many countries [1]. Supplementation with these *n*-3 PUFAs may also be beneficial for a wide range of other noncommunicable diseases (NCDs), including cancer, metabolic diseases, arthritis and neurodegenerative conditions [2].

However, the mechanism(s) by which EPA and DHA, as well as more complex *n*-3 PUFA oil mixtures, impact on wide-ranging human pathologies remains poorly delineated. An overarching concept is that EPA and DHA are anti-inflammatory, acting directly on target tissues or indirectly by modulation of the host immune response. This may occur via inhibition of pro-inflammatory lipid mediator signalling controlled by cyclooxygenases and lipoxygenases and/or secondary to production of so-called specialised pro-resolving mediators (SPMs) such as resolvins, protectins and maresins [2].

With the explosion of interest and published work on the role of gut microbiota on pathogenesis of diverse NCDs, including cancer, neurodegenerative conditions such as Parkinson's disease and Alzheimer's disease, psychiatric illness, including bipolar disorder, and metabolic disorders including metabolic-associated steatotic liver disease [3–5], research has been directed towards investigation of whether some or all of the beneficial effects of *n*-3 PUFAs are explained by modulation of gut microbiota.

Initial studies in rodent models and humans demonstrated that dietary and pharmacological supplementation with *n*-3 PUFAs was associated with a small but consistent shift in the gut microbiome in favour of bacteria predicted to generate short-chain fatty acids (SCFAs) [6,7], which are thought to underlie beneficial effects on gut mucosal integrity and immune-regulation [8,9^▪^].

Herein, we review latest findings that are beginning to uncover a causal link between gut microbiota changes and benefits from *n*-3 PUFA intake in animal models, that further define the changes to the faecal microbiome associated with *n*-3 PUFA use in humans, and that are starting to shed light on how oral intake of n-3 PUFAs leads to changes to the gut microbiome.

Latest data on faecal bacterial microbiota changes are associated with oral n-3 PUFA intake. 

**Box 1:**
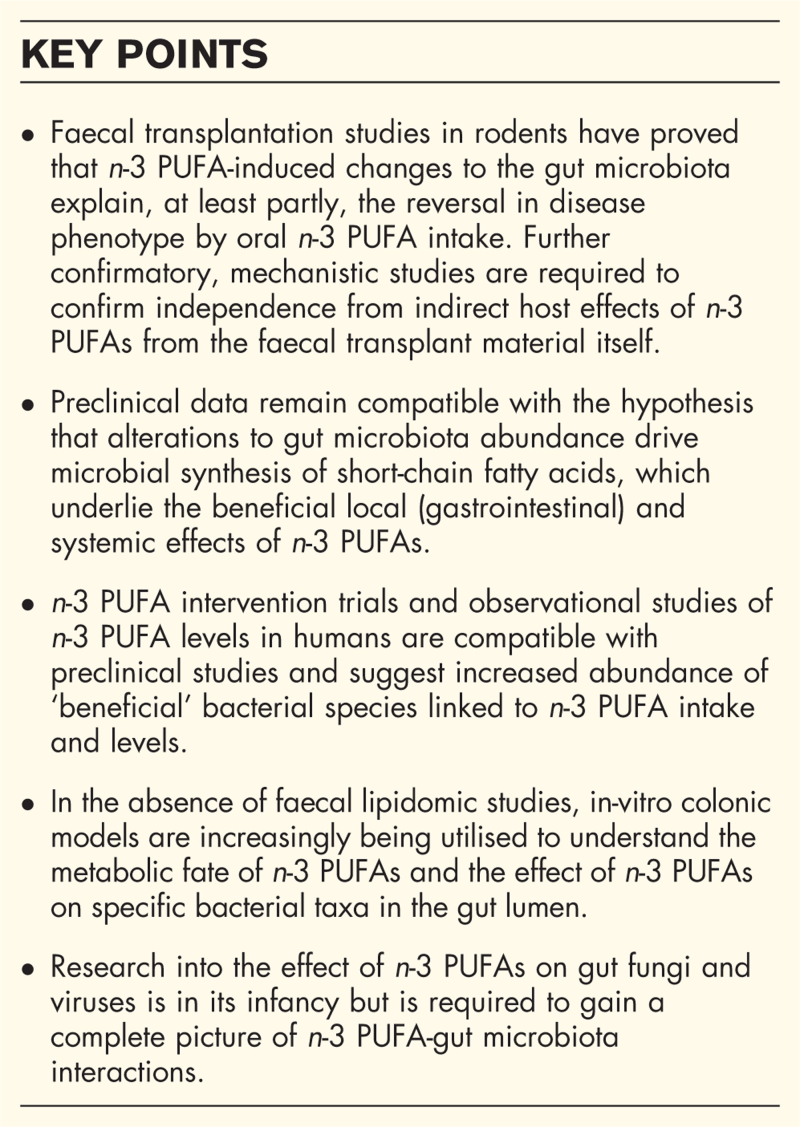
no caption available

## RODENT STUDIES

A systematic review published in 2025 described 32 preclinical studies in which the effect of a *n*-3 PUFA intervention (including complex fish oil or perilla oil *n*-3 PUFA mixtures, or purified EPA and DHA formulations) on the faecal microbiota profile was investigated in animals receiving a high-fat diet (HFD) driving diet-induced obesity (DIO) [10^▪▪^]. All the individual studies that were identified up until mid-2025 used 16S rRNA characterisation of the faecal microbiome [10^▪▪^]. Despite marked heterogeneity in study design, including the quantity and mixture of *n*-3 PUFAs in the diet, there was consistent reversal of the ‘dysbiosis’ associated with the HFD with a decrease in the *Firmicutes*/*Bacteroidetes* (F/B) ratio in the presence of dietary *n*-3 PUFA supplementation [10^▪▪^]. There was also a study-specific increase in abundance of ‘beneficial’ species including *Akkermansia muciniphila* and reduced abundance of taxa considered to be pro-inflammatory such as *Desulfovibrio *[10^▪▪^]. Similar observations have been reported by Li *et al.* [11] using a flaxseed (rich in *n*-3 PUFA alpha-linolenic acid [ALA]) intervention in a mouse HFD-induced obesity model. A clear limitation of these studies that investigated the effect of *n*-3 PUFA supplementation in the context of obesity-related dysbiosis has been almost complete reliance on a single mouse genetic background (C57Bl/6) precluding an understanding of the effect of oral *n*-3 PUFA intake in mouse models with different genetic backgrounds that display differential host immune system profiles. In a single study using male Jcl:ICR mice, a perilla seed oil (rich in ALA) intervention (and control fish oil intervention) abrogated the reduction in microbial diversity that accompanied azoxymethane-dextran sodium sulphate-induced intestinal tumorigenesis [12].

The systematic review of studies, which included reports dating back to 2016, highlighted that there is no consistent effect of dietary *n*-3 PUFAs on alpha- or beta-diversity of the HFD-associated faecal microbiome [10^▪▪^]. The results of a 2025 study by Portela *et al.* [13] concurred with this conclusion and importantly provided data on the lack of any dose-dependency of the changes to the gut microbiome related to proportion of *n*-3 PUFAs in the diet of male C57Bl/6 mice fed a HFD. This study also delineated an increase in regulatory T cells in visceral adipose tissue associated with dietary *n*-3 PUFA supplementation in a DIO model [13].

Bourragat and colleagues [14,15^▪^] recently extended their previous study of protection from DIO by transplantation of caecal microbiota of *fat-1* transgenic mice, which have high tissue levels of *n*-3 PUFAs. Their latest caecal microbiota transplantation experiments (this time, performed in the absence of antibiotic pretreatment of recipient wild-type mice) confirmed that *fat-1* caecal microbiota provided partial protection against DIO and glycaemic control in wild-type mouse recipients and decreased colonic mucus disruption, but did affect lipopolysaccharide endotoxaemia [15^▪^]. More detailed microbiome analysis demonstrated that *fat-1* microbiota transplantation was associated with increased abundance of *Bilophila* and genera from the *Ruminococcaceae* family predicted to be SCFA producers [15^▪^]. Overall, these important studies confirm a causal relationship between altered gut microbiota associated with high host tissue *n*-3 PUFA levels and the beneficial effects of *n*-3 PUFAs on the DIO phenotype. Huang *et al*. [16] used a rat model of acne vulgaris and reported that transplantation of the gut microbiota from animals exposed to *n*-3 PUFAs by oral gavage to untreated recipient rats decreased experimental acne endpoints. Further gut microbiota transplantation studies after oral supplementation with *n*-3 PUFAs in rodent models are awaited to confirm that protection from disease phenotypes by oral intake of *n*-3 PUFAs is explained, at least partly, by changes to the gut microbiota, as opposed to direct systemic effects of the *n*-3 PUFAs themselves. Of note, the published faecal transplant studies have not ruled out systemic effects of *n*-3 PUFAs transferred from the transplant inoculum itself [15^▪^,^16^].

Modulation of mouse gut microbiota by *n*-3 PUFA supplementation has also been studied in the context of other human disease models. In contrast to mouse DIO models (predominantly using male animals), female transgenic APP/PS1 mice (a model of Alzheimer's disease) receiving a relatively small amount (0.3% w/w in chow) of dietary EPA displayed an increase in abundance of *Firmicutes* and decrease in *Bacteroidetes* phyla (the opposite of changes to the F/B ratio in DIO models) compared with animals receiving control chow for 3 weeks [17]. Moreover, both fish oil and a microalgal supplement (rich in EPA) were associated with an increase in the F/B ratio in a rat CCl4-induced liver fibrosis model [18]. The difference in gut microbiota changes in DIO models compared with other disease models highlights the likely importance of background diet on changes to the mouse gut microbiome associated with *n*-3 PUFA supplementation. Treatment with EPA has also been shown to reduce prostate tumour cell growth and confer changes to the gut microbiota (with a prominent reduction in *Ruminococcaceae* family abundance) in C57Bl/6 mice [19].

Several other recent reports have detailed the effect of a *n*-3 PUFA intervention in combination with another agent in a rodent model, including a plant extract (in a mouse D-galactose accelerated ageing model) and vitamin D (in a rat DIO model) [20,21].

## HUMAN STUDIES

Relatively few human studies have been performed, to date. Of note, the OCEAN randomised, placebo-controlled trial of 3360 mg mixed EPA and DHA ethyl esters daily for 12 weeks in 309 Chinese participants with type 2 diabetes and hypertriglyceridaemia included detailed characterisation of the faecal microbiome by metagenomic sequencing, as a secondary outcome [22^▪▪^]. Consistent with the findings from DIO models in rodents, there were no significant changes in alpha or beta-diversity during the trial [22^▪▪^]. There were also no clear differences in individual taxa compared with findings prior to the intervention in participants randomised to either *n*-3 PUFAs or corn oil placebo [22^▪▪^]. However, the baseline faecal microbiota profile predicted the triglyceride-lowering effect of *n*-3 PUFAs more strongly than either baseline clinical phenotype or fasting serum lipid profile [22^▪▪^].

Another human faecal microbiome study of oral *n*-3 PUFA supplementation published in the previous 18 months was a crossover study in which 35 healthy adults were randomised to receive either a combination of fermentable fibre and high-dose (7.7 g per day) fish oil for 30 days or its comparator (maltodextrin and corn oil) for 30 days, separated by a 60-day washout period before each participant received the other intervention [23]. This study revealed differences in the faecal microbiome between the two interventions in individual volunteers (beta-diversity) [23], similar to a previous crossover trial of *n*-3 PUFA treatment in healthy volunteers [7], with the caveat that the individual contributions of the fibre and *n*-3 PUFA components of the intervention could not be distinguished by Moosavi *et al*. [23]. These studies highlight the ability of the crossover trial design to detect small intra-individual changes in the faecal microbiome associated with *n*-3 PUFA supplementation, on a background of much larger variability in the gut microbiota that exists between individuals. Consistent with previous data [7], 16S rRNA sequencing revealed an increase in abundance of taxa predicted to synthesise SCFAs associated with the combination fibre and *n*-3 PUFA intervention [23]. Huang *et al*. [16] reported a randomised trial of mixed *n*-3 PUFAs (2.4 g daily for 12 weeks) versus no added treatment in 46 isotretinoin-treated acne vulgaris patients that described changes to the gut microbiota at the end of the treatment period that included an increase in microbiota diversity between individuals, as well as increased abundance of *Coprococcus*, *Eubacterium* and *Intestinibacter* genera, compared with baseline findings.

Another noteworthy human observational study of 250 Chinese mother-infant pairs reported that a high breast milk *n*-6 PUFA arachidonic acid concentration (and, conversely, low EPA and DHA concentration) were associated with gut microbiota ‘dysbiosis’ and increased risk of atopic dermatitis in infants [24^▪^]. Accompanying mechanistic studies demonstrated that intra-gastric arachidonic acid administration exacerbated atopic dermatitis-like skin inflammation and gut microbiota dysbiosis in mice, but that EPA administration did not abrogate skin inflammation compared with control animals, which did not receive PUFA treatment [24^▪^]. A smaller observational breast-feeding mother-infant study of the gut microbiome related to gestational diabetes revealed an inverse relationship between gut bacterial phyla abundance (including Acidobacteriota and Gemmatimonadota) and breast milk total *n*-3 PUFAs levels [25].

## IN-VITRO COLONIC MODELS

In-vitro colonic (also known as faecal fermentation) models facilitate detailed investigation of the time-course and dose-dependency of the effect of *n*-3 PUFAs on the faecal microbiota, albeit with potential limitations related to accurate modelling of colonic physiology and luminal bioavailability of nutrients in both static (single reaction chamber) and dynamic (multiple linked reaction chambers mimicking regional differences in physiology) models [26]. Salsinha *et al*. [27] used a static faecal fermentation model and gastrointestinal digestion protocol (in order to mimic prior passage of nutrients through the gastrointestinal tract prior to colonic luminal exposure) to test the effect of menhaden fish oil and a concentrated EPA and DHA formulation on selected bacterial taxa in fermentation reactions derived from five healthy human donors [27], following earlier work using rat faecal fermentations [28]. During 48-h fermentations, there were only small changes in bacterial abundance in fish oil and EPA/DHA reactions (containing effective EPA and DHA concentrations between 10 and 40 μg/μl) compared with the changes observed with the fructooligosaccharide control, with the most prominent increase in abundance observed for *Akkermansia* species at 24 h [27]. Roussel and colleagues have reported the use of the dynamic Simulator of the Human Intestinal Microbial Ecosystem (SHIME) model to study the effects of mixed EPA and DHA triglycerides (*n* = 1 donor) [29], and subsequently of *Buglossoides arvensis* oil, which is rich in *n*-3 PUFA stearidonic acid (*n* = 4 donors), on microbial communities along the modelled GI tract [30]. The SHIME model highlighted the increase in abundance of *Akkermansia muciniphila* associated with addition of EPA and DHA to the model, in a gastrointestinal tract location (transverse and descending colon vessels) and niche (luminal mucin-associated)-dependent manner [29]. Similar findings were associated with the presence of *Buglossoides arvensis* oil [30]. Both interventions were associated with a small, specific increase in the concentration of SCFA propionate at the end of the 7-day test period [29,30]. Similar findings linked to the presence of EPA and DHA (in separate reactions), with elevated SCFA concentrations associated with an increased F/B ration and increased abundance of bacterial taxa including *Veillonella*, were reported in an independent SHIME model experiment that used faeces from only one donor [31].

## NON-BACTERIAL GUT MICROBIOTA

In a novel study, Xiao *et al.* [32^▪^] investigated the relationship between the abundance of faecal fungal genera, identified from a discovery cohort (*n* = 764) by internal transcribed spacer (ITS) 2 sequencing [33], and plasma *n*-3 PUFA levels in three other small, disparate validation cohorts [32^▪^]. Four fungal genera were inversely associated with plasma total *n*-3 PUFA levels [32^▪^]. Secondary observations revealed the relationship between individual fungal genera abundance and both glycaemic control and diagnosis of type 2 diabetes [32^▪^]. Another study has reported changes in the gut mycobiome associated with oral administration (by gavage) of EPA and DHA ethyl esters in male C57Bl/6 mice, alongside gut bacterial abundance measured by 16S rRNA sequencing [34]. Exposure to oral *n*-3 PUFAs was associated with reduced abundance of potentially pathogenic fungal taxa *Aspergillus* and *Penicillium *[34]. Correlation analysis suggested a relationship between abundance of specific bacterial and fungal taxa but stopped short of proof of any causal bacterial-fungal relationship [34]. These preliminary observational data on a possible link between *n*-3 PUFA status and the gut mycobiome should stimulate further investigation of a potential role for this small, but important, component of the gut microbiota in mediating the health benefits of *n*-3 PUFAs [9^▪^].

The authors are unaware of any published work on the relationship between *n*-3 PUFAs, the gut virome and human disease despite increased understanding of the interplay between the gut viruses, bacterial components of the microbiota and disease pathogenesis [35]. This is clearly an important area for future research.

## FAECAL METABOLOMIC CHANGES ASSOCIATED WITH *n*-3 PUFAs

**FIGURE 1:**
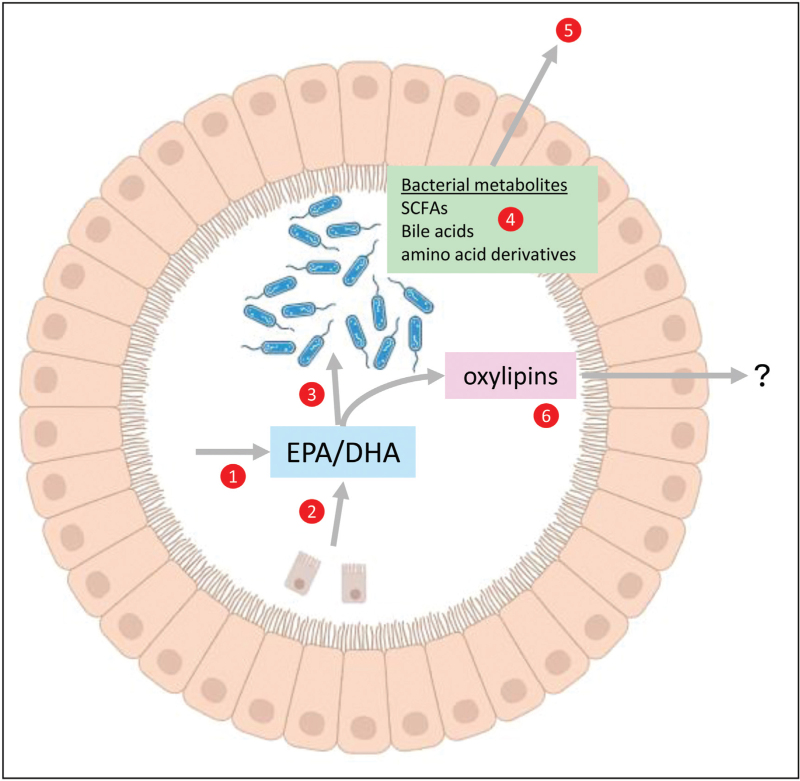
Interplay between luminal *n*-3 PUFAs, the intestinal microbiota and the bacterial/host metabolome. The two main bioactive *n*-3 PUFAs EPA and DHA are bioavailable in the intestinal lumen via dietary (mainly oily fish) and/or nutritional supplement use, if they have avoided absorption in the proximal small intestine (1). Alternatively, EPA and DHA that have already been systemically absorbed and then incorporated in tissues may become bioavailable in the GI tract lumen through enterocyte shedding and breakdown (2). It is not known whether de-novo synthesis of EPA and DHA by gut microbiota occurs from shorter-chain *n*-3 PUFAs including alpha-linolenic acid. Evidence from in-vitro colonic models and descriptive studies of the faecal microbiome in humans suggests that *n*-3 PUFAs alter the abundance of bacterial taxa (3), thereby modifying the intestinal bacterial metabolome (4). A valid hypothesis is that at least some of the health benefits of *n*-3 PUFAs in the human host are explained by systemic absorption and activity of bacterial metabolites such as SCFAs, including butyrate, and amino acid metabolites (5). EPA and DHA could also be metabolised by bacteria to oxylipins that may have local intestinal activity and/or act systemically after absorption (6). Created in https://BioRender.com.

After initial characterisation of the effect of *n*-3 PUFA intake on the gut microbiota composition in animal models and in humans, attention is now increasingly turning to investigation of accompanying faecal metabolomic changes that may underlie the beneficial effects of *n*-3 PUFAs on gut microbiota composition (Fig. 1). Experiments have focussed on colonic SCFA production consistent with the notion that SCFAs, particularly propionate and butyrate, modulate the immune response [8] and have neuroprotective properties [36], in addition to undoubted local effects on the gut mucosal barrier [9^▪^,^37^]. Direct SCFA measurements in in-vitro models and bacterial metagenomic data from human studies are consistent with promotion of colonic SCFA production as an important consequence of gut microbiota changes induced by oral *n*-3 PUFA intake [23,29]. An important area for future health research is to determine whether combined intake of dietary fibres (the substrates for bacterial SCFA production) can augment the effect of *n*-3 PUFAs on the abundance of gut bacteria that produce SCFAs.

Recent reviews have highlighted the significant contribution of many gut bacterial metabolites to the host metabolome [38,39]. Other gut microbial metabolites that may be impacted by the alteration in gut microbiota abundance associated with *n*-3 PUFA use include trimethylamine-N-oxide (TMAO) and secondary bile acid metabolites, which are considered to be deleterious for cardiovascular health [4,9^▪^].

The effect of the gut microbiota on host tissue PUFA content and oxylipin levels has yet to be delineated (Fig. 1). Further work is required to understand a potential link between the gut microbiota (and probiotic interventions) and mammalian cell PUFA levels that was initially described over 15 years ago [40].

## METABOLISM OF *n*-3 PUFAs BY GUT MICROBIOTA

In-vitro colonic models have recently provided new insights into the metabolic fate of EPA and DHA that are present in the colon. The concentration of most fatty acids (including *n*-3 PUFAs) decreased in a time-dependent manner over 48 h in a static fermentation model [27]. However, EPA and DHA free fatty acid (FFA) levels in the vessels that represent different regions of the distal GI tract in the SHIME model tended to increase over time, which the authors suggested was due to FFA release from triglycerides occurring in the modelled proximal gastrointestinal tract [29].

Data from the in-vitro SHIME model have suggested that EPA and DHA undergo limited metabolism to monohydroxylated oxylipins such as 15-HEPE and 18-HEPE without downstream metabolism to other bioactive oxylipins such as resolvins [41]. Detailed lipidomic studies of intestinal content throughout the length of the gastrointestinal tract are required to understand the metabolic fate of *n*-3 PUFAs inside the gastrointestinal lumen and the relationship of microbiota-derived oxylipins to the circulating and tissue lipidome in humans (Fig. 1).

Some gut bacterial species, including *Bifidobacterium* species, can convert *n*-6 PUFA linoleic acid to conjugated isomers termed conjugated linoleic acid (CLA), which have experimental anticancer activity [42,43]. It is currently unclear whether bacterial metabolism of *n*-3 ALA to conjugated linolenic acids is relevant to the bioactivity of oils that contain significant amounts of ALA.

## HOW DO *n*-3 PUFAS ALTER THE GUT MICROBIAL PROFILE AND/OR BACTERIAL METABOLOME?

Despite an increasing body of observational evidence that oral *n*-3 PUFA administration is associated with changes to rodent and human gut microbiota composition and metabolic activity, there is only rudimentary understanding of how *n*-3 PUFAs have these effects (Fig. 1). Central to understanding mechanism(s) is delineation of whether *n*-3 PUFAs act directly on bacterial populations in the gut lumen (entering the distal gut lumen after incomplete proximal absorption of orally administered n-3 PUFAs or secondary to enterocyte shedding after systemic absorption and tissue uptake) as opposed to indirectly via modulation of host gastrointestinal mucosal immunity-microbiota interplay (Fig. 1). Careful kinetic tracer studies and specific gastrointestinal *n*-3 PUFA delivery methods (bypassing physiological upper small bowel absorption of *n*-3 PUFAs) will be required to understand the origin of luminal *n*-3 PUFAs and complex interplay between *n*-3 PUFAs and multiple bacterial taxa *in vivo*. Better understanding of the origin and levels of individual *n*-3 PUFAs in the lumen of different parts of the gastrointestinal tract will facilitate design of studies that determine exactly how PUFAs interact with the gut microbiota, building on historical data from the 1970's onwards, as well as a more recent report, that *n*-3 PUFA FFAs have direct bacterio-static/cidal properties *in vitro*[44].

## CONCLUSION

Evidence continues to build from rodent models, in-vitro experiments using human faecal samples, and human observational studies that oral *n*-3 PUFA intake is associated with changes to the gut microbiota that are predicted to be beneficial for health. Currently, evidence that the gut microbiota contributes to the health benefits attributed to *n*-3 PUFAs, as opposed to systemic activity of *n*-3 PUFAs following gastrointestinal absorption, is limited to faecal transplant experiments in rodents. It remains unclear how *n*-3 PUFAs affect changes to the gut microbiota or whether *n*-3 PUFA metabolism by gut microbes contributes to the host metabolome. Therefore, evidence currently falls short of that needed to define *n*-3 PUFAs as prebiotic according to the working definition of the International Scientific Association of Probiotics and Prebiotics (ISAPP), ‘a substrate that is selectively utilised by host microorganisms conferring a health benefit’ (https://isappscience.org/for-scientists/resources/prebiotics/, accessed 27 August 2025). Future research priorities include work to understand how *n*-3 PUFAs selectively alter gut microbiota composition, and comparison of the effect of oral *n*-3 PUFA delivery on faecal and host metabolomes, including PUFA and oxylipin profiles.

## Acknowledgements

*None.*

### Financial support and sponsorship

*M.A.H. is a Co-Investigator of the National Institutes of Health grant R01CA243454 (Prebiotic effect of eicosapentaenoic acid treatment for colorectal cancer liver metastasis) which is studying the effect of EPA treatment on the faecal microbiome during the Yorkshire Cancer Research-funded (RA/2015/R2/004) EMT2 trial (NCT032428477). The work carried out to prepare this narrative review was independent of the above grants.*

### Conflicts of interest

*M.A.H. receives grants from Yorkshire Cancer Research (RA/2015/R2/004) and NIH (R01CA243454) which investigate n-3 PUFAs. These grants are not linked to this manuscript. H.S. declares no conflict of Interest.*

## REFERENCES AND RECOMMENDED READING

Papers of particular interest, published within the annual period of review, have been highlighted as:

▪ of special interest
▪▪ of outstanding interest

## References

[1] Dong S Wang Y Bian J. The effect of omega-3 polyunsaturated fatty acid (PUFA) prescription preparations on the prevention of clinical cardiovascular disease: a meta-analysis of RCTs. Nutr J 2024; 23:157. 39639295 10.1186/s12937-024-01051-y PMC11622672

[2] Xu R Molenaar AJ Chen Z Yuan Y. Mode and mechanism of action of omega-3 and omega-6 unsaturated fatty acids in chronic diseases. Nutrients 2025; 17:1540. 40362847 10.3390/nu17091540 PMC12073370

[3] Xie M Li X Lau HC Yu J. The gut microbiota in cancer immunity and immunotherapy. Cell Mol Immunol 2025; 22:1012–1031. 40770084 10.1038/s41423-025-01326-2 PMC12398524

[4] Muttiah B Hanafiah A. Gut microbiota and cardiovascular diseases: unravelling the role of dysbiosis and microbial metabolites. Int J Mol Sci 2025; 26:4264. 40362500 10.3390/ijms26094264 PMC12072866

[5] Balakrishnan R Kang SI Lee JY. Gut microbiota-immune system interactions in health and neurodegenerative diseases: insights into molecular mechanisms and therapeutic applications. Aging Dis 2024; doi: 10.14336/AD.2024.1362. 10.14336/AD.2024.1362 PMC12539548 39656490

[6] Caesar R Tremaroli V Kovatcheva-Datchary P. Crosstalk between gut microbiota and dietary lipids aggravates WAT inflammation through TLR signaling. Cell Metab 2015; 22:658–668. 26321659 10.1016/j.cmet.2015.07.026 PMC4598654

[7] Watson H Mitra S Croden FC. A randomised trial of the effect of omega-3 polyunsaturated fatty acid supplements on the human intestinal microbiota. Gut 2018; 67:1974–1983. 28951525 10.1136/gutjnl-2017-314968

[8] Mann ER Lam YK Uhlig HH. Short-chain fatty acids: linking diet, the microbiome and immunity. Nat Rev Immunol 2024; 24:577–595. 38565643 10.1038/s41577-024-01014-8

[9▪] Ross FC Patangia D Grimaud G. The interplay between diet and the gut microbiome: implications for health and disease. Nat Rev Microbiol 2024; 22:671–686. 39009882 10.1038/s41579-024-01068-4

[10▪▪] Cao B Sun Y Lam C. Interaction between dietary omega-3 polyunsaturated fatty acids, obesity and gut microbiota in preclinical models: a systematic review of randomized controlled trials. Diabetes Obes Metab 2025; 27:4643–4661. 40536121 10.1111/dom.16535

[11] Li H Shen M Chen X. Dietary flaxseed oil and its blended oil alleviate high-fat diet-induced obesity in mice by improving lipid metabolism and regulating gut microbiota. Foods 2025; 14:1877. 40509405 10.3390/foods14111877 PMC12155290

[12] Korsirikoon C Techaniyom P Kettawan A. Cold-pressed extraction of perilla seed oil enriched with alpha-linolenic acid mitigates tumour progression and restores gut microbial homeostasis in the AOM/DSS mice model of colitis-associated colorectal cancer. PLoS One 2024; 19:e0315172. 39652552 10.1371/journal.pone.0315172 PMC11627366

[13] Portela ND Eberhardt N Bergero G. Dietary omega-3 supplementation shapes gut microbiota and regulates immunometabolism in a mouse model of obesity. Med Res Arch 2025; 13. doi: 10.18103/mra.v13i5.6623.

[14] Bidu C Escoula Q Bellenger S. The transplantation of ω3 PUFA-altered gut microbiota of fat-1 mice to wild-type littermates prevents obesity and associated metabolic disorders. Diabetes 2018; 67:1512–1523. 29793999 10.2337/db17-1488

[15▪] Bourragat A Escoula Q Bellenger S. The transplantation of the gut microbiome of fat-1 mice protects against colonic mucus layer disruption and endoplasmic reticulum stress induced by high fat diet. Gut Microbes 2024; 16:2356270. 38797998 10.1080/19490976.2024.2356270 PMC11135845

[16] Huang Y Liu F Lai J. The adjuvant treatment role of ω-3 fatty acids by regulating gut microbiota positively in the acne vulgaris. J Dermatolog Treat 2024; 35:2299107. 38164791 10.1080/09546634.2023.2299107

[17] Altendorfer B Benedetti A Mrowetz H. Omega-3 EPA supplementation shapes the gut microbiota composition and reduces major histocompatibility complex class II in aged wild-type and APP/PS1 Alzheimer's mice: a pilot experimental study. Nutrients 2025; 17:1108. 40218866 10.3390/nu17071108 PMC11990804

[18] Uthaiah NM Venkataramareddy SR Mudhol S Sheikh AY. EPA-rich Nannochloropsis oceanica biomass regulates gut microbiota, alleviates inflammation and ameliorates liver fibrosis in rats. Food Res Int 2025; 202:115733. 39967180 10.1016/j.foodres.2025.115733

[19] Lachance G Robitaille K Laaraj J. The gut microbiome-prostate cancer crosstalk is modulated by dietary polyunsaturated long-chain fatty acids. Nat Commun 2024; 15:3431. 38654015 10.1038/s41467-024-45332-w PMC11039720

[20] Martin M Boulaire M Lucas C. Plant extracts and ω-3 improve short-term memory and modulate the microbiota-gut-brain axis in D-galactose model mice. J Nutr 2024; 154:3704–3717. 39332773 10.1016/j.tjnut.2024.09.015

[21] Le Jan D Siliman Misha M Destrumelle S. Omega-3 fatty acid and vitamin D supplementations partially reversed metabolic disorders and restored gut microbiota in obese Wistar rats. Biology (Basel) 2024; 13:1070. 39765737 10.3390/biology13121070 PMC11673857

[22▪▪] Lu J Liu R Ren H. Impact of omega-3 fatty acids on hypertriglyceridemia, lipidomics, and gut microbiome in patients with type 2 diabetes. Med 2025; 6:100496. 39163858 10.1016/j.medj.2024.07.024

[23] Moosavi D Mullens DA Davidson LA. Gut microbial community and host intestinal gene expression with combined fish oil and soluble corn fiber compared with corn oil and maltodextrin: a randomized crossover trial in healthy older individuals. Am J Clin Nutr 2025; 122:396–412. 40754387 10.1016/j.ajcnut.2025.04.031 PMC12405781

[24▪] Jiang S Cai M Li D. Association of breast milk-derived arachidonic acid-induced infant gut dysbiosis with the onset of atopic dermatitis. Gut 2024; 74:45–57. 39084687 10.1136/gutjnl-2024-332407 PMC11671956

[25] Li K Jin J Liu Z. Dysbiosis of infant gut microbiota is related to the altered fatty acid composition of human milk from mothers with gestational diabetes mellitus: a prospective cohort study. Gut Microbes 2025; 17:2455789. 39834317 10.1080/19490976.2025.2455789 PMC11776479

[26] Isenring J Bircher L Geirnaert A Lacroix C. In vitro human gut microbiota fermentation models: opportunities, challenges, pitfalls. Microbiome Res Rep 2023; 2:2. 38045607 10.20517/mrr.2022.15 PMC10688811

[27] Salsinha AS Araújo-Rodrigues H Dias C. Omega-3 and conjugated fatty acids impact on human microbiota modulation using an in vitro fecal fermentation model. Clin Nutr 2025; 49:102–117. 40262394 10.1016/j.clnu.2025.04.007

[28] Salsinha AS Cima A Araújo-Rodrigues H. The use of an *in vitro* fecal fermentation model to uncover the beneficial role of omega-3 and punicic acid in gut microbiota alterations induced by a Western diet. Food Funct 2024; 15:6095–6117. 38757812 10.1039/d4fo00727a

[29] Roussel C Anunciação Braga Guebara S Plante PL. Short-term supplementation with ω-3 polyunsaturated fatty acids modulates primarily mucolytic species from the gut luminal mucin niche in a human fermentation system. Gut Microbes 2022; 14:2120344. 36109831 10.1080/19490976.2022.2120344 PMC9481098

[30] Roussel C Sola M Lessard-Lord J. Human gut microbiota and their production of endocannabinoid-like mediators are directly affected by a dietary oil. Gut Microbes 2024; 16:2335879. 38695302 10.1080/19490976.2024.2335879 PMC11067990

[31] Rehman A Pham V Seifert N. The polyunsaturated fatty acids eicosapentaenoic acid and docosahexaenoic acid, and vitamin K_1_ modulate the gut microbiome: a study using an in vitro Shime model. J Diet Suppl 2024; 21:135–153. 37078491 10.1080/19390211.2023.2198007

[32▪] Xiao C Xi Y Wang X. Association of plasma n-3 polyunsaturated fatty acid with gut mycobiome and implications for glucose homeostasis. Sci China Life Sci 2025. 10.1007/s11427-024-2850-y 40516019

[33] Shuai M Fu Y Zhong H. Mapping the human gut mycobiome in middle-aged and elderly adults: multiomics insights and implications for host metabolic health. Gut 2022; 71:1812–1820. 35017200 10.1136/gutjnl-2021-326298 PMC9380515

[34] Chai Z Zhang H Ji X. The disparate effects of omega-3 PUFAs on intestinal microbial homeostasis in experimental rodents under physiological condition. Prostaglandins Leukot Essent Fatty Acids 2024; 203:102643. 39317024 10.1016/j.plefa.2024.102643

[35] Cao Z Sugimura N Burgermeister E. The gut virome: a new microbiome component in health and disease. EBioMedicine 2022; 81:104113. 35753153 10.1016/j.ebiom.2022.104113 PMC9240800

[36] Jabbari Shiadeh SM Chan WK Rasmusson S. Bidirectional crosstalk between the gut microbiota and cellular compartments of brain: implications for neurodevelopmental and neuropsychiatric disorders. Transl Psychiatry 2025; 15:278. 40796700 10.1038/s41398-025-03504-2 PMC12343879

[37] Smolinska S Popescu FD Zemelka-Wiacek M. A review of the influence of prebiotics, probiotics, synbiotics, and postbiotics on the human gut microbiome and intestinal integrity. J Clin Med 2025; 14:3673. 40507435 10.3390/jcm14113673 PMC12156228

[38] Van Treuren W Dodd D. Microbial contribution to the human metabolome: implications for health and disease. Annu Rev Pathol 2020; 15:345–369. 31622559 10.1146/annurev-pathol-020117-043559 PMC7678725

[39] Jyoti Dey P. Mechanisms and implications of the gut microbial modulation of intestinal metabolic processes. NPJ Metab Health Dis 2025; 3:24. 40604123 10.1038/s44324-025-00066-1 PMC12441142

[40] Wall R Ross RP Shanahan F. Impact of administered bifidobacterium on murine host fatty acid composition. Lipids 2010; 45:429–436. 20405232 10.1007/s11745-010-3410-7

[41] Roussel C Lessard-Lord J Nallabelli N. Human gut microbes produce EPA- and DHA-derived oxylipins, but not N-acyl-ethanolamines, from fish oil. FASEB J 2025; 39:e70713. 40515551 10.1096/fj.202500752RR

[42] González A Fullaondo A Rodríguez J. Conjugated linoleic acid metabolite impact in colorectal cancer: a potential microbiome-based precision nutrition approach. Nutr Rev 2025; 83:e602–e614. 38728013 10.1093/nutrit/nuae046 PMC11723137

[43] Chen Y Fang H Chen H. Bifidobacterium inhibits the progression of colorectal tumorigenesis in mice through fatty acid isomerization and gut microbiota modulation. Gut Microbes 2025; 17:2464945. 39924893 10.1080/19490976.2025.2464945 PMC11812354

[44] Waller ME Gutierrez A Ticer TD. Profiling the response of individual gut microbes to free fatty acids (FFAs) found in human milk. J Funct Foods 2025; 125:106664. 40051690 10.1016/j.jff.2025.106664 PMC11884519

